# Mechanisms of inclusion of thallium-201 into Prussian blue nanoparticles for nuclear medicine applications†

**DOI:** 10.1039/d4tb01203h

**Published:** 2024-07-08

**Authors:** Katarzyna M. Wulfmeier, Philip J. Blower, Galo Paez Fajardo, Steven Huband, Rafael T. M. de Rosales, David Walker, Samantha YA Terry, Vincenzo Abbate, Juan Pellico

**Affiliations:** a School of Biomedical Engineering and Imaging Sciences, King's College London London UK juan.pellico@kcl.ac.uk; b Warwick Manufacturing Group, University of Warwick UK; c Department of Physics, University of Warwick UK; d Institute of Pharmaceutical Sciences, King's College London UK

## Abstract

Prussian blue is known for its high affinity for thallium and other univalent metal cations and has been used as a treatment for radiocaesium and thallium/radiothallium poisoning. While Prussian blue nanoparticles (PBNPs) show potential for binding radioactive thallium for further use in nuclear medicine applications, the inclusion mechanism remains elusive. Understanding the interaction between PBNPs and ^201^Tl is essential for identifying the physicochemical and radiochemical properties required for optimal *in vivo* performance. In this work, we evaluated the binding mechanism between Tl and PBNPs with different coatings and core shapes. Combining PBNPs with [^201^Tl] thallium(i) chloride provided high radiolabelling yields and radiochemical stabilities under physiological conditions. Comprehensive characterisation by different X-ray techniques confirmed that Tl ions are located in the interstitial sites within the crystal structure, maintaining the integrity of the iron (Fe) 4p electronic distribution and inducing local modifications in the nearby C–N ligands. Additionally, this inclusion does not impact the core or the shell of the nanoparticles but does alter their ionic composition. The PB ionic network undergoes significant changes, with a substantial drop in K^+^ content, confirming that Tl^+^ ions replace K^+^ and occupy additional spaces within the crystal structure. These results open new opportunities in nuclear medicine applications with ^201^Tl-PBNPs where the size, shape and composition of the particles can be specifically tuned depending on the desired biological properties without affecting the radiochemical performance as a vehicle for ^201^Tl.

## Abstract 2

Prussian blue nanoparticles are radiolabelled with Tl-201 and characterised to provide the unambiguous confirmation of the inclusion mechanism.

## Graphical abstract

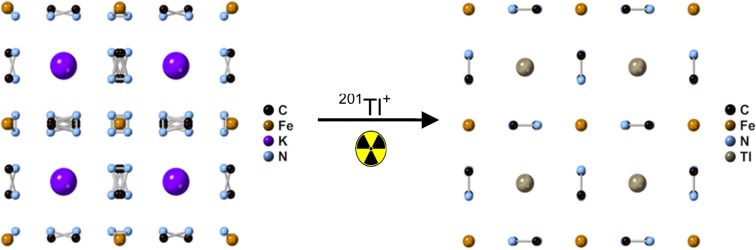

## Introduction

Thallium-201 (^201^Tl, *t*_1/2_ = 73 h) is an Auger electron-emitting radionuclide with high potential for radionuclide therapy due to its high linear energy transfer (LET) and short-range electron emissions.^1–3^ Over the years, this radionuclide has been used in the form of [^201^Tl]TlCl as a radiotracer for myocardial perfusion in single-photon emission computed tomography (SPECT).^4,5^ However, despite its remarkable radiotoxic properties, its application in molecular radiotherapy has been hindered by the absence of efficient and stable chelators capable of delivering ^201^Tl to specific target tissues.^2,6^ Consequently, extensive efforts have been dedicated to searching for effective ^201^Tl chelators in recent years,^6,7^ so far without success. An important advance was recently reported by Rigby *et al.*, who successfully labelled branched polydentate picolinic acid-based chelators (H_4_pypa, H_5_decapa, H_4_neunpa-NH_2_, and H_4_noneunpa) with ^201^Tl(iii).^7^ These compounds, chosen for their multidentate N- and O-donor groups, had previously demonstrated high efficacy with radionuclides such as ^111^In, ^177^Lu, and ^225^Ac. Although these ligands represented a significant improvement over traditional DOTA and DTPA chelators, their radiochemical stability with ^201^Tl, a crucial factor for targeted radiotherapy, was insufficient for *in vivo* radiotherapeutic use.

The lack of suitable molecular chelators for ^201^Tl has spurred the exploration of alternative methods to establish stable conjugates. One such promising alternative involves the use of nanoparticles as effective carriers for delivering radionuclides to tumour cells. Precise control of the physicochemical properties of nanoparticles, along with the ability to customise their surface coating, allows for tailored design for specific biomedical applications. Using nanoparticles for radionuclide delivery, whether for diagnostic or therapeutic purposes, offers significant advantages, including the ability to modulate the pharmacokinetics, stability and solubility, and to control radionuclide release.

**Fig. 1:**
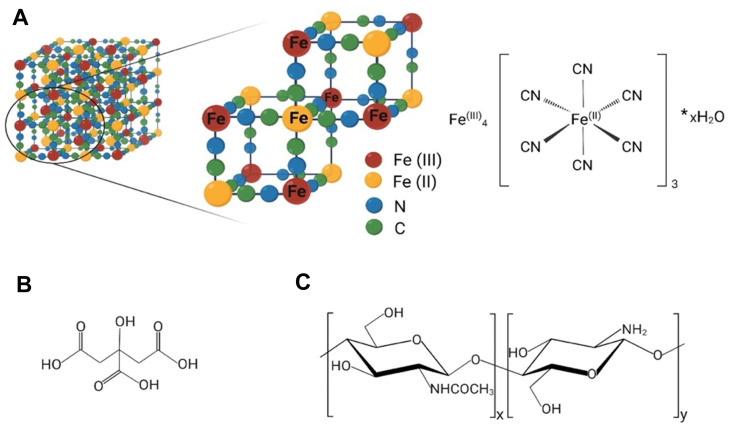
(A) The simplified octahedral crystal structure and structural formula of Prussian blue. For simplicity, water molecules coordinated to Fe(ii) sites (coordinative water) and the water molecules inside interstitial cavities (zeolitic water) are not shown. (B) Chemical structure of citric acid (2-hydroxy-1,2,3-propanetricarboxylic acid). (C) Chemical structure of chitosan; the structure consists of *N*-acetyl-2-amino-2-deoxy-d-glucopyranose (acetylated unit) and 2-amino-2-deoxy-d-glucopyranose (deacetylated unit), where the repeating units are linked by β-(1 → 4)-glycosidic bonds. Created with BioRender.

Prussian blue (PB) is a well-established treatment for radiocaesium (^137^Cs) poisoning and, under the name Radiogardase®, it has also been approved by the US Food and Drug Administration (FDA) for radioactive/non-radioactive thallium poisoning.^8^ PB, also known as ferric hexacyanoferrate(ii), with empirical formula Fe^(iii)^_4_[Fe^(ii)^(CN)_6_]_3_·*x*H_2_O (*x* = 14–16), was initially synthesised at the beginning of 18th century for use as a dye. Soluble PB can form colloids in aqueous conditions and has a formula represented as AFe^(iii)^_4_[Fe^(ii)^(CN)_6_]_3_·*x*H_2_O, where *x* = 1–5 and A is a monovalent cation, such as K^+^, Na^+^ or NH_4_^+^.^9^ In 1936, Keggin and Miles studied PB for the first time using X-ray diffraction, discovering its face-centred structure with a calculated 10.2 Å cubic-cell dimension and proposing a model where ferric and ferrous atoms were located at the corners of the cubic lattice with the cyanide groups forming the edges and univalent metals like potassium presumed to occupy the octahedral interstitial lattice sites (Fig. 1A).^10^

In the years that followed the initial discovery, a substantial amount of data gathered using X-ray diffraction analysis, infrared and Mössbauer spectroscopy, and photoelectron spectroscopy led to the emergence of more comprehensive models, including confirmation that the presence of potassium or other alkali metals within the PB crystal structure does not impact the oxidation state of iron atoms.^11^ Despite this knowledge and the well-recognised affinity of Tl^+^ ions for PB, particularly in clinical applications, the mechanism of binding of univalent cations remains unclear. It has been suggested that the mechanisms could include adsorption, entrapment or ion exchange but conclusive evidence is lacking.^12,13^

Prussian blue nanoparticles (PBNPs) coated with citric acid were first synthesised by Shokouhimehr *et al.* for potential use as magnetic resonance imaging (MRI) contrast agents.^14^ Subsequently, Szigeti *et al.* successfully radiolabelled these nanoparticles with ^201^Tl, enabling dual MRI/SPECT imaging.^15,16^ The *in vivo* biodistribution of radioactive PB-bound ^201^Tl can be significantly altered by modifying the size, composition, and surface properties of PBNPs.^15,17,18^ Nevertheless, without knowledge of the mechanism by which ^201^Tl interacts with PBNPs, the potential rational design of these parameters to control *in vivo* behaviour is limited. In this study, we synthesised PBNPs with cubic and spherical shapes and coated them with two compounds imparting opposite surface charges: citric acid and chitosan (Fig. 1B and C). We evaluated their radiolabelling performance with ^201^Tl^+^ and conducted a comprehensive characterisation using multiple X-ray techniques. This approach provided the first unambiguous confirmation of the binding mechanism between thallium and PBNPs.

## Results

### Physicochemical characterisation of Prussian blue nanoparticles

**Fig. 2:**
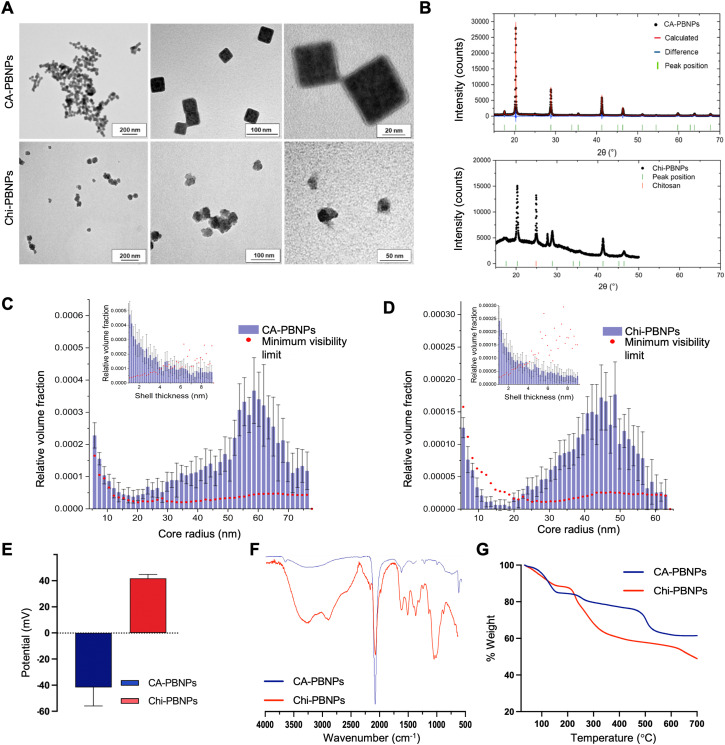
(A) Transmission electron microscopy (TEM) images of the citric-acid coated Prussian blue nanoparticles (CA-PBNPs, top row) and chitosan-coated Prussian blue nanoparticles (Chi-PBNPs, bottom row) at different magnifications. TEM analysis revealed cubic-shaped crystals for CA-PBNPs with an average diameter of 47.1 ± 11.5 nm calculated over 506 particles, while Chi-PBNPs showed an irregular spherical shape with average diameter of 57.6 ± 27.3 nm calculated over 276 particles, (B) X-ray diffraction (XRD) spectra for CA-PBNPs (top) and Chi-PBNPs (bottom) showing characteristic peaks consistent with *Fm*3̄*m* PB crystal structure. (C) SAXS measurements for CA-PBNPs presenting average core radius of 48.4 ± 19.2 nm and average shell thickness of 3.8 ± 2.3 nm; (D) SAXS measurements for Chi-PBNPs; average core radius of 39.1 ± 14.2 nm and shell thickness: 3.7 ± 2.2 nm; (E) ζ-Potential measurements for CA-PBNPs (*n* = 10) and Chi-PBNPs (*n* = 6) showing a negative value of −41.7 ± 14.2 mV for CA-PBNPs and a positive value of 41.8 ± 3.0 mV for Chi-PBNPs; (F) Fourier-transformed infrared spectra (FTIR) of CA-PBNPs (blue) and Chi-PBNPs (red) between 4000 and 500 cm^−1^ with peaks characteristic for PB and specific coatings; (G) thermogravimetric analysis (TGA) from 25 °C to 800 °C of CA-PBNPs and Chi-PBNPs confirming the presence of different coatings.

PBNPs were synthesised following a straightforward method involving mixing either FeCl_3_ with K_4_[Fe(CN)]_6_ or FeCl_2_ with K_3_Fe[(CN)]_6_ in the presence of citric acid (CA-PBNPs) or chitosan (Chi-PBNPs) used as coating to ensure formation of colloidally stable suspensions. The size distribution and shape of CA-PBNPs and Chi-PBNPs were determined by transmission electron microscopy (TEM). The TEM images of CA-PBNPs revealed cubic shaped crystals with an average diameter of 47.1 ± 11.5 nm (*n* = 506), while TEM images of Chi-PBNPs showed an irregular spherical shape with average diameter of 57.6 nm ± 27.3 (*n* = 276) (Fig. 2A). X-ray diffraction (XRD) measurements confirmed the structure of the particles as Prussian blue (Fig. 2B). While both types of particles exhibited similar patterns, Chi-PBNPs showed an additional peak at 24.89° due the presence of chitosan, along with an unidentified peak at 27.7°.

A more detailed analysis by small-angle X-ray scattering (SAXS) showed an average core radius of 48.4 ± 19.2 nm for CA-PBNPs and 39.1 ± 14.2 nm for Chi-PBNPs whilst the average shell thickness was 3.8 ± 2.3 nm and 3.7 ± 2.2 nm, respectively (Fig. 2C and D and Table S1, ESI†). Further experiments were conducted to evaluate the coating of the nanoparticles. First, ζ-potential measurements showed a negative value of −41.7 ± 14.2 mV for CA-PBNPs and a positive value of 41.8 ± 3.0 mV for Chi-PBNPs (Fig. 2E). The UV-visible absorption spectra for both types of nanoparticles showed a broad absorption band from 600 to 850 nm with a maximum around 700 nm, characteristic of Prussian blue (Fig. S1, ESI†). Fourier-transformed infrared spectra of both types of particles revealed a strong band at 2062 cm^−1^ corresponding to the C≡N stretching vibration of the Fe^2+^–CN–Fe^3+^ in the crystal lattice characteristic of Prussian blue, as well as bands at 604 cm^−1^ and 502 cm^−1^ attributed to the Fe^2+^–CN vibration (Fig. 2F). Moreover, the spectrum of CA-PBNPs showed the asymmetric and symmetric carboxyl stretching 1605 and 1396 cm^−1^ bands corresponding to the citric acid. The Chi-PBNPs sample showed bands spanning from 3188 to 3324 cm^−1^, indicating N–H, O–H stretching and intramolecular hydrogen bonds. Furthermore, bands at 2924 and 2883 cm^−1^ were attributed to C–H symmetric and asymmetric stretching typical for polysaccharides; bands at 1621 and 1377 cm^−1^ belonged to the *N*-acetyl groups, bands at 1516 cm^−1^ corresponded to the N–H bending and bands at 1060 and 1025 cm^−1^ due to the C–O stretching, confirmed the presence of chitosan in the coating of the particles. Finally, thermogravimetric analysis (TGA) showed a 34.4% mass lost in a 3-step manner typically found in citric acid-coated nanoparticles for CA-PBNPs and a 45.5% of mass lost for Chi-PBNPs in a 2-step manner commonly found in chitosan-coated PBNPs (Fig. 2G).^19,20^ These data allowed the estimation of the number of coating molecules per particle, providing 8.1 × 10^4^ citric acid molecules per particle for CA-PBNPs and 143 chitosan molecules per particle for Chi-P BNPs (Table S2, ESI†).

### Radiolabelling of Prussian blue nanoparticles with ^201^Tl

**Fig. 3:**
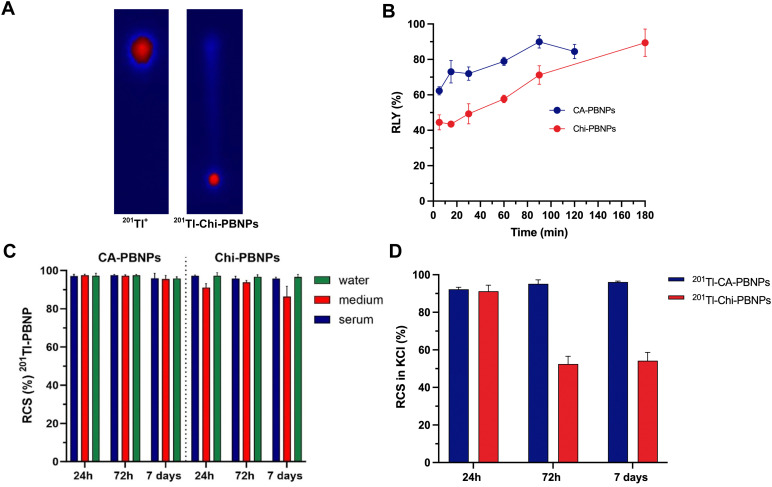
(A) Example of radio-TLC plate imaged with phosphor imager of [^201^Tl]TlCl (left) and ^201^Tl-Chi-PBNPs (right) at room temperature. Mobile phase: 10 mM EDTA solution; (B) radiolabelling yield (%RLY) 5 to 180 min after incubation of CA-PBNPs at 0.5 mg mL^−1^ and Chi-PBNPs at 0.25 mg mL^−1^ with 30 kBq of ^201^Tl at room temperature. RLY was determined by radio-TLC before the purification process, *n* = 3; (C) radiochemical stability (%RCS) of ^201^Tl-CA-PBNPs and ^201^Tl-Ch-PBNPs after 24 h, 72 h and 7 days incubation in water, RPMI medium and human serum at 37 °C, *n* = 3; (D) radiochemical stability (%RCS) of ^201^Tl-CA-PBNPs and ^201^Tl-Chi-PBNPs after 24 h, 72 h and 7 days in 25 mM KCl solution at room temperature, *n* = 3.

The radiolabelling of the nanoparticles with ^201^Tl was evaluated using radio-thin layer chromatography (radio-TLC). A solution of EDTA (10 mM) was employed as a mobile phase moving free ^201^Tl^+^ to the front (*R*_f_ = 0.9) while the radiolabelled particles remain at the origin (*R*_f_ = 0) (Fig. 3A). In the case of CA-PBNPs, the radiolabelling yield (RLY) increased from 62.3 ± 2.3% after 5 min to 84.5 ± 4.1% after 120 min. Lower RLY was observed for Chi-PBNPs at the early time points (44.5 ± 4.2% after 5 min). However, an increase in the incubation time to 180 min provided a 89.4 ± 7.7% RLY (Fig. 3B). The radiochemical stability (RCS) of both samples was tested in water, tissue culture medium and serum. High values of RCS are essential to avoid the release of free radionuclide *in vivo*. CA-PBNPs showed high RCS values in all three conditions (95.8 ± 1.0% at 37 °C after 7 days in serum). Chi-PBNPs demonstrated similar behaviour in serum (96.6 ± 1.4% at 37 °C after 7 days, Fig. 3C). Further stability tests in the presence of 25 mM KCl were conducted to explore potential transmetalation reactions. The results showed stabilities higher than 90% for both samples after 24 h. At 72 h a significant decrease to 52.5 ± 4.1% was observed for Chi-PBNPs, in contrast to CA-PBNPs which remained stable after 7 days (Fig. 3D).

### Characterisation of Tl^+^ inclusion into PBNPs

**Fig. 4:**
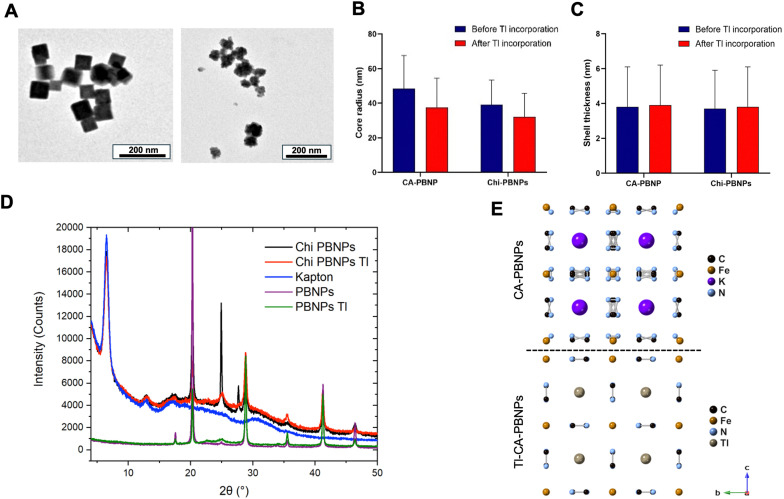
(A) Transmission electron microscopy (TEM) images of Tl^+^-doped Prussian blue nanoparticles coated with citric acid (Tl-CA-PBNPs, left) and with chitosan (Tl-Chi-PBNPs, right) (see Fig. 2A for comparison with Tl-free particles). TEM images revealed no significant changes in shape or size compared to the non-doped samples; (B) average core radius (nm) obtained from the small-angle X-ray scattering (SAXS) fitting data for CA-PBNPs, Chi-PBNPs, Tl-CA-PBNPS and Tl-Chi-PBNPs; (C) Average shell thickness (nm) obtained from the small-angle X-ray scattering (SAXS) fitting data for CA-PBNPs, Chi-PBNPs, Tl-CA-PBNPS and Tl-Chi-PBNPs. SAXS measurements showed that core radius and shell thickness for both of PBNPs was not affected by Tl incorporation; (D) X-ray diffraction (XRD) spectra for CA-PBNPs, Chi-PBNPs, Tl-CA-PBNPs and Tl-Chi-PBNPs. XRD results showed a large impact of thallium loading on the diffraction intensities for both samples; (E) schematic representation of the crystal structure for CA-PBNPs (top) and Tl-CA-PBNPs (bottom) obtained by Rietveld refinement of the XRD data which confirmed that Tl^+^ is located in interstitial spaces within the crystal structure of the nanoparticles. For CA-PBNPs the refinements were obtained on the assumption that the interstitial spaces are occupied (*e.g.*, by potassium).

Physicochemical characterisation of the nanoparticles after incubation with non-radioactive thallium (Tl^+^) was carried out to determine the nature of the interaction between the metal ion and the PBNPs. TEM imaging revealed no significant changes in shape and size compared to the non-thallium samples, with a measured size of 46.1 ± 11.2 nm for Tl-CA-PBNPs and 59.4 ± 27.3 nm for Tl-Chi-PBNPs (Fig. 4A). SAXS measurements showed that core radius and shell thickness for both samples were unaffected by Tl incorporation (Fig. 4B and C and Table S1, ESI†). However, XRD studies showed a large impact of thallium loading on the diffraction pattern for both samples (Fig. 4D). Rietveld refinement of the data for Tl-CA-PBNPs confirmed that Tl^+^ is located in interstitial spaces within the crystal structure of the nanoparticles (Fig. 4E).

**Fig. 5:**
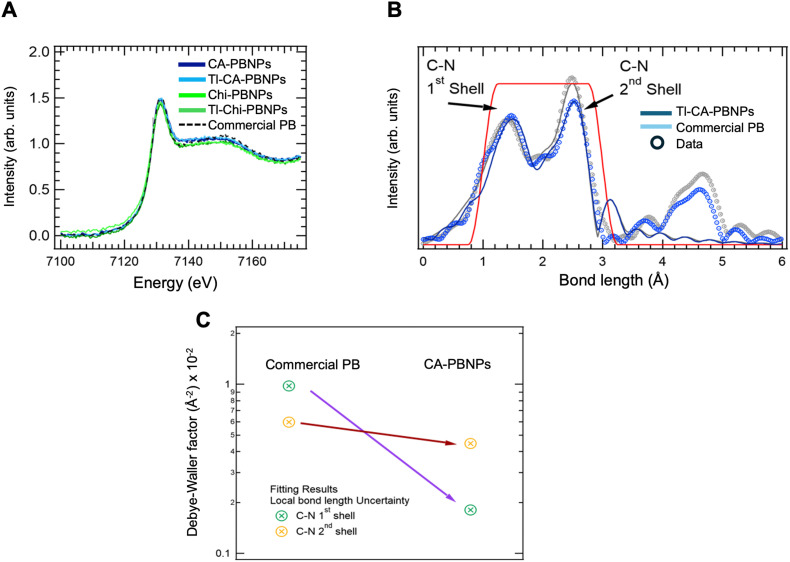
(A) X-ray absorption near edge structure (XANES, Fe-K edge) of CA-PBNPs, Chi-PBNPs, Tl-CA-PBNPs, Tl-Chi-PBNPs and commercial Prussian blue (PB) used as reference (Sigma-Aldrich). The comparison revealed no significant differences between Tl-free and Tl-doped samples and confirmed that the electronic integrity of the crystal structure is not affected by the incorporation of Tl^+^; (B) X-ray absorption fine structure (EXAFS) K-edge data and fitting of Tl-CA-PBNPs and the reference PB (Sigma-Aldrich). The fitting results indicated that the N atoms of the CN ligand are located nearer to the Fe^3+^ and the C atoms closer to the Fe^2+^ ions. The red window indicates the bond length domain used in the fitting; (C) Debye–Waller (DW) factors from the fitting results of the EXAFS data for Tl-CA-PBNPs and the reference PB (Sigma-Aldrich). The differences observed for 1st and 2nd shell confirmed that Tl ions are placed in interstitial spaces of the crystal structure inducing small local changes of the C–N ligands rather than in the Fe 4p electron distribution.

A more detailed investigation was carried out by Fe K-edge X-ray Absorption (XANES), which produced a scan sensitive to the oxidation states of Fe and the local octahedral environment (Fig. 5A). A comparison of CA-PBNPs and Chi-PBNPs, both with and without thallium, revealed no significant differences between Tl-free and Tl-doped samples. This confirms that the oxidation state of Fe ions, and hence the electronic integrity of the crystal structure, is not affected by the incorporation of Tl^+^ (Fig. 5A and Fig. S2, ESI†). Additionally, we used an uncoated commercial PB sample to evaluate whether the coatings affect the integrity of the crystal structure. The results showed no significant modifications, confirming that the coatings do not alter the oxidation state of the Fe ions.

We performed extended X-ray absorption fine structure (EXAFS) spectroscopy to evaluate the ionic local geometry around the Fe atoms (Fig. 5B). The first peak, corresponding to the first shell, was attributed to the two different CN octahedra present in PB materials whilst the second peak corresponded to C and N atoms around the Fe in next-nearest oxidation environments. The fitting results indicated that the N atoms of the CN ligand are located nearer to the Fe^3+^ and the C atoms closer to the Fe^2+^ ions. Further, Debye–Waller (DW) factors of the EXAFS fitting results revealed significant differences between the commercially available PB crystals used as a reference and the Tl-doped CA-PBNPs both first and second shells. These differences confirm that Tl ions are placed in interstitial spaces of the crystal structure inducing small local changes of the C–N ligands rather than in the Fe 4p electron distribution (Fig. 5C).

**Fig. 6:**
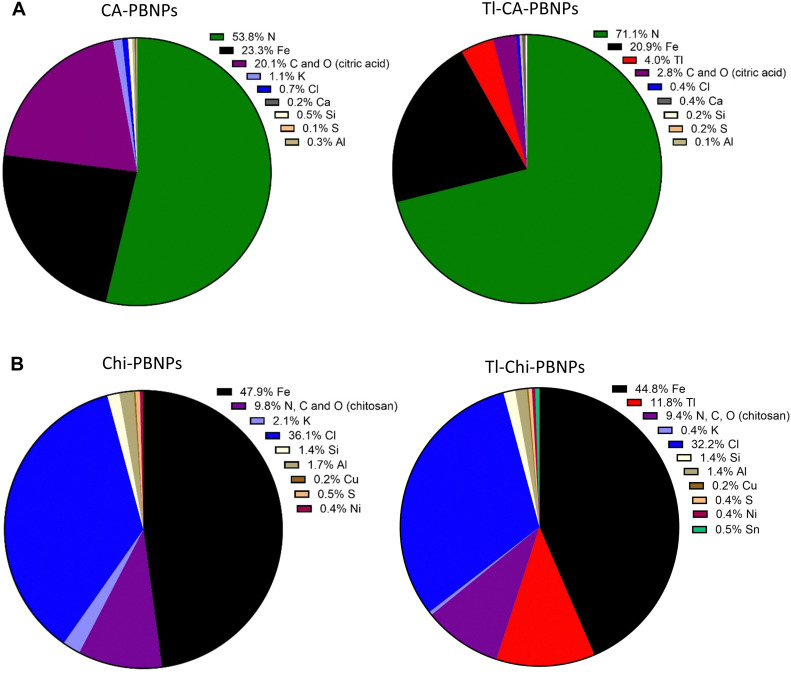
(A) Elemental analysis by X-ray fluorescence (XRF) of CA-PBNPs (left) and Tl doped CA-PBNPs (Tl-CA-PBNPs, right); (B) XRF of Chi-PBNPs (left) and Tl doped Chi-PBNPs (Tl-Chi-PBNPs, right). Data are expressed as molar percentage (% molar composition). The results indicate that thallium was efficiently incorporated into the crystal structure. Initially, the amount of potassium within the PB crystal structure was low in both types of non-doped PBNPs and decreased significantly after Tl incorporation. Chi-PBNPs contained significantly more Cl ions than CA-PBNPs. Higher amounts of Si detected in all samples are related to the sample preparation method. The molar percentage for C and O present in citric acid coating and N, C and O present in chitosan coating were calculated taking into account the elemental content of these elements in Prussian blue and cellulose, which was used to form pellets in the preparation method.

Finally, X-ray fluorescence (XRF) was used to examine the elemental composition of both types of PB nanoparticles, pre- and post-incubation with TlCl (Fig. 6, Table 1 and Fig. S3, ESI†). It was assumed that the Fe content, representing the amount of PB in the nanoparticles, remained constant during Tl doping. Therefore, changes in molar composition are primarily expressed relative to the Fe content. (Table 1). Molar composition was chosen as a more effective illustration of those changes than mass percentage obtained directly using XRF. The majority of the molar content in CA-PBNPs and Chi-PBNPs comprised Fe, N, C, and O, reflecting the crystal structure and coatings. The amount of potassium detected in the PB crystal structure was relatively low in both types of non-doped PBNPs (1.1% for CA-PBNPs and 2.1% for Chi-PBNPs, Fig. 6), and both decreased after the Tl incorporation to 0.4% in Chi-PBNPs and below the detection limit in case of CA-PBNPs. This resulted in a five-fold increase in the Fe : K molar ratio for Chi-PBNPs and a 29-fold increase for CA-PBNPs (Table 1). Thallium was effectively integrated into the PB crystal structure, with a ratio of 1 mole of Tl for every 45 moles of Fe in both PBNP types. A similar Tl : Fe molar ratio of 1 : 5 was confirmed for Tl-CA-PBNPs using an alternative analytical method (Table S3, ESI†). Other elements detected included aluminium, calcium and sulfur, with a total of 0.6% molar content in both non-doped and doped CA-PBNPs, and aluminium, calcium, sulfur, nickel and tin, with a 2.8% of molar composition in non-doped Chi-PBNPs and 2.4% in Tl-doped Chi-PBNPs. The presence of silicon, ranging from 0.2–1.4%, was attributed to the sample preparation method. Chi-PBNPs contained significantly more Cl ions than CA-PBNPs (36.1% *vs.* 0.7%, respectively), which decreased to 32.2% for Tl-Chi-PBNPs and to 0.4% for Tl-CA-PBNPs. The molar composition of commercially available PB is presented in Fig. S4 (ESI†). In this sample, no potassium was detected; instead, sodium was found, comprising 13.8% of the molar content.

**Table 1:** Molar ratios of the elements of interest in non-doped and thallium-doped CA-PBNPs and Chi-PBNPs. Calculations are based on X-ray fluorescence (XRF) analysis. Thallium was effectively integrated into the PB crystal structure, with a ratio of 1 mole of Tl for every 4–5 moles of Fe in both PBNP types. Tl incorporation resulted in a fivefold increase in the Fe : K molar ratio for Chi-PBNPs and a 29-fold increase for CA-PBNPs

	Molar ratios			
	CA-PBNPs	Tl-CA-PBNPs	Chi-PBNPs	Tl-Chi-PBNPs
Fe : K	20.9	597.6	23.0	115.8
Fe : Cl	35.2	51.9	1.3	1.4
Cl : K	0.6	11.5	17.3	83.3
Fe : Tl	—	5.2	—	3.8
Tl : K	—	114.7	—	30.6
Tl : Cl	—	10.0	—	0.4

## Discussion

We have synthesised PBNPs with two different coatings and core shapes to assess their efficacy in radiolabelling with ^201^Tl. First, we obtained CA-PBNPs by co-precipitation of citric acid mixed with K_4_[Fe(CN)_6_] and citric acid combined with FeCl_3_. In this method, the carboxylic acid groups of the citric acid complex the ferric ions to form a precursor that controls the nucleation of the particles while at the same time coating their surface.^14^ Due to its simplicity, this one-step aqueous synthesis is very common and provides colloidal stable PBNPs of different sizes, with cubic shape. Different strategies have emerged in the last years to develop non-cubic, sphere-like particles.^21,22^ In this work, we have modified a reported protocol using chitosan as template to control the growth of the particles.^20^ TEM images showed that our method produces sphere-like Chi-PBNPs of similar size as the CA-PBNPs but in a more irregular shape. In the synthesis, Fe(CN)_6_^3−^ ions are mixed with chitosan prior to the addition of Fe^2+^. The irregular shape can be attributed to the random distribution of these ions along the chitosan macromolecules triggering different crystal growth when in contact with Fe^2+^. Both CA-PBNPs and Chi-PBNPs showed high stability and no aggregates, and the XRD measurements confirmed their PB crystal structure. The disparity in size observed in TEM and SAXS measurements for both, CA-PBNPs and Chi-PBNPs, is a result of the different types of distributions provided by these two methods. Specifically, TEM analysis provides size measurements based on a number distribution, whereas SAXS analysis relies on a volume distribution. Furthermore, as expected, compared to CA-PBNPs, Chi-PBNPs exhibited a larger particle shell due to the macromolecular nature of chitosan, and opposite surface charge corresponding to the presence of protonated NH_3_^+^ instead of the COO^−^ in CA-PBNPs.

The radiolabelling of the nanoparticles yielded high RLY regardless the core shape and surface composition. Chi-PBNPs required longer incubation times than CA-PBNPs to reach RLYs over 85%. This might be related to the high molecular weight of chitosan providing steric hindrance for ^201^Tl^+^ to access the core of the particles, or the electrostatic barrier to thallium cations imposed by the positive surface charge. More importantly, both labelled particles exhibit an RCS higher than 95% after incubation in human serum at 37 °C for 7 days.

In terms of particle stability, Doveri *et al.* demonstrated a rapid degradation of the PB network at pH levels above 7 in uncoated particles due to the formation of hydroxocomplexes from labile Fe^3+^ coordination sites.^23^ In our study, both types of radiolabelled nanoparticles remained stable at room temperature, likely due to the presence of the coating agents, for up to 72 hours in water, medium and human serum, with no discoloration observed. In combination, the high stability of the radiolabelling and the particles under physiological conditions predict a suitable performance in potential targeted radiotherapy applications where the ^201^Tl is required to remain attached to the particle until reaching the region of interest. However, nearly a 40% reduction in the RCS of Tl-Chi-PBNPs after incubation in KCl solution for 3 to 7 days suggests that prolonged exposure to high concentrations of K^+^ can lead to partial transmetalation and the release of thallium. This process is likely to occur intracellularly, where K^+^ concentrations rise to 150 mM.^24^ The high RCS in human serum indicates that transmetalation does not occur extracellularly, where K+ concentration is limited to 3.5–5 mM, making these particles suitable for *in vivo* applications.^25^

A deeper evaluation of the radiolabelling mechanism reveals that incorporation of Tl^+^ affects the general properties of neither the core nor the shell of the particles significantly. This is important since the physicochemical properties of the particles must remain unchanged after the radiolabelling to avoid issues in further *in vivo* applications. The diffraction patterns confirm that Tl^+^ is located within the interstitial spaces of the crystal structure. Using the Rietveld method as a model to refine the data, we were able to determine the amount of Tl^+^ doping required to model the data and calculate the atomic positions within the particles (Fig. 4E). Detailed structural investigations and Rietveld refinement were challenging for Tl-Ch-PBNPs due to the physical presentation of the sample. The use of Kapton sheets for measurements resulted in a strong background signal, which made the structural refinements difficult. Therefore, the analysis of Tl-Chi-PBNPs relied on a qualitative comparison of the similar changes in the diffraction pattern observed for Tl-CA-PBNPs. Furthermore, XANES and EXAFS techniques were performed to evaluate the effects of the Tl^+^ doping in the electronic network. By using Fe K-edge X-ray absorption, the electronic properties of Fe-based compounds can be measured *via* the dipole Fe 1s to 4p transition.^26^ This dipole transition is sensitive to the Fe oxidation state and increases with it. The fact that the intensity and the shift in the K-edge absorption energy remain identical (Fig. 5) for the Tl-free and Tl-doped samples confirms that the presence of Tl^+^ in the interstitial spaces does not alter the oxidation state of the Fe ions and the electronic network is not affected, which in turn confirms that Tl^+^ is in the *Fm*3̄*m* interstitial spaces. Additionally, the samples follow the same pattern as a commercial non-coated PB sample indicating that the coatings are located at the surface of the particle and do not interact in the inclusion of Tl^+^. The local coordination of Tl^+^ within the crystal structure was evaluated *via* the photoelectron wavelength inter-ion scattering interference using EXAFS. According to the model, the first Fe shell corresponds to two different C–N octahedra due to the two different Fe oxidation states (Fe^2+^ and Fe^3+^) in PB while the second Fe shell corresponds to C and N ions interacting with nearby Fe^2+^ and Fe^3+^. Fitting the results for the DW factors reveals that the doping with Tl^+^ decreases the first shell DW factor at an exponential scale factor despite the inherent uncertainties due to the different Fe oxidation states, while the second shell factor remains similar. Hence, the incorporation of Tl^+^ makes the CN ligands shift slightly closer to the Fe^2+^ ions in a manner that seeks to homogenise the electronic distribution around the Fe^2+^ ions.

As expected, the molar composition of the nanoparticles undergoes significant changes following thallium doping. XRF revealed a relatively low amount of potassium in both types of PBNPs’ crystal structures, suggesting that not every interstitial space is occupied (Fe : K molar ratios of 21 : 1 for CA-PBNPs and 23 : 1 for Chi-PBNPs). The substantial drop in the potassium content (as expressed by increased Fe : K ratios) after Tl incorporation indicates that potassium vacates its interstitial spaces, making them available for thallium. The Fe to Tl molar ratio was calculated as 5 : 1 for CA-PBNPs and 4 : 1 for Chi-PBNPs, which is much lower compared to potassium, suggesting that thallium occupies more interstitial spaces than potassium. This indicates that thallium replaces potassium in the interstitial spaces and additionally occupies new ones. Furthermore, Chi-PBNPs contain around 35 times more chlorine than CA-PBNPs, likely due to chlorine being present on the surface of Chi-PBNPs balancing the positive charge of amine groups in chitosan. Considering the negatively charged citric acid coating, we assume that Cl is only present in the CA-PBNPs crystal structure at the amount of 0.7%. It is possible that iron binds chloride and other anions at the coordination sites not occupied by cyanide, given that the coordination sites of iron are not saturated in the non-doped PBNPs structure. Chloride might also be present in the interstitial spaces, in close proximity to positively charged ions like potassium or thallium. The increasing Cl : K ratio in both types of nanoparticles, as potassium leaves the crystal structure after Tl incorporation, suggests that chloride may act as a counterion to potassium but not balancing the total amount of incorporated thallium. This is evidenced by the Tl : Cl molar ratio for CA-PBNPs calculated as 10 : 1. However, based on the XRF results, it remains challenging to determine what ions (hydroxide perhaps) counterbalances the increased positive charge of thallium incorporated into the crystal structure.

The radiolabelling studies show that ^201^Tl^+^ binds to PB nanoparticles efficiently by occupying the interstitial spaces within the crystal structure regardless of the coating used, and this integration remains stable under physiological conditions. Following functionalisation, PBNPs can thus serve as highly efficient carriers for delivering radioactive Tl^+^ to cancer cells, enabling targeted molecular radiotherapy. Therefore, the combination of PBNPs with ^201^Tl^+^ offers an attractive alternative to chelators based on small-size molecules. Importantly, the use of the nanoparticles eliminates the requirement to oxidise Tl(i) to Tl(iii) required by small molecular chelators. This not only simplifies the radiolabelling but mitigates the risk of Tl being released form the binding complex due to the facile reverse reduction in physiological conditions, as observed in a previous study.^7^

## Conclusions

Two different types of Prussian blue nanoparticles coated with citric acid and chitosan were successfully synthesised. These nanoparticles have different shapes and opposite surface charges while sharing similarities in size, crystal structure and ability to include thallium(i) ions. Radiolabelling of both types of PBNPs proved to be efficient, resulting in radiochemically stable nanoparticles under diverse conditions, including biological media, up to 72 h. The mechanism of thallium inclusion entails thallium ions occupying interstitial spaces in the PB lattice, and inclusion does not impact the core or shell of the nanoparticles, but it does affect their ionic composition. The ionic network undergoes significant modifications, with a substantial drop in K^+^ content confirming that Tl^+^ ions replace K^+^ and also occupy additional spaces within the crystal structure. Importantly, it is often assumed that the stability of metal doped nanoparticles is achieved by their interaction with the metallic electronic network. Our results indicate that Tl^+^ ions occupying interstitial sites do not disrupt the Fe 4p electron distribution but do induce small, localised changes in the relative positions of CN ligands. Overall, the successful and stable binding of radioactive thallium by PBNPs presents a promising avenue for the targeted therapeutic delivery of ^201^Tl, regardless of the size and shape of the particles. This introduces a range of possibilities, wherein distinct PBNPs can be employed to access specific tissues of interest based on their unique physicochemical properties without affecting the radiotherapeutic performance.

## Materials and methods

All chemicals, unless specified, were purchased from Sigma-Aldrich, UK. [^201^Tl]TlCl in sterile 0.9% NaCl solution (280–580 MBq/5.8 mL) was obtained from Curium Pharma, France.

### Synthesis of citric-acid coated PBNPs (CA-PBNPs)

The synthetic method and purification process of citric acid coated Prussian blue nanoparticles (CA-PBNPs) were adapted and optimised from a previously published work.^15^ In brief, 105 mg of citric acid (as monohydrate, 0.5 mmol) was added to 20 mL of 1 mM anhydrous FeCl_3_. The solution was heated to 60–65 °C and then 20 mL of 1 mM K_4_[Fe(CN)_6_]·3H_2_O (Alfa Aesar) with 105 mg citric acid was added dropwise over 10 min. The suspension was left for another minute stirring at 60–65 °C and then cooled down. To purify the nanoparticles, 40 mL of the CA-PBNPs suspension was moved to Amicon® filter tubes (15 mL, 30 000 MWCO) and centrifuged at 4200 rpm for 10 min (Rotina 380R, Hettich). The filtrate solution was discarded and CA-PBNPs were resuspended with MilliQ water and centrifuged again. This process was repeated twice. CA-PBNPs were re-suspended in 5 mL of MilliQ water (concentration approximately 1 mg mL^−1^); pH: 6–7. To obtain dry powder, the CA-PBNPs aqueous suspension was freeze-dried at −54 °C, 0.1 mbar (LTE Scientific™ Freeze Dryer Lyotrap).

### Synthesis for chitosan coated PBNPs (Chi-PBNPs)

A chitosan solution (0.1 mg mL^−1^ in 0.5 M HCl) was stirred for 1 h at room temperature and filtered through a 0.45 μm filter. Then, 5 mL of 1 mM K_3_Fe(CN)_6_ aqueous solution was added to 20 mL of the chitosan solution at room temperature (RT) while stirring. After 30 min, 5 mL of 1 mM FeCl_2_·4H_2_O was added dropwise and the mixture was stirred for 1 hour at RT. Finally, 50 mL of acetone was added and particles were collected by centrifugation at 4000 rpm for 10 min, washed with a mixture of 0.5 M HCl and 0.5 M acetone (20 : 80 v/v) three times, collected by centrifugation and dried under vacuum for 24 h.^27^

### Incubation with non-radioactive thallium

2.5 mL of either CA-PBNPs or Chi-PBNPs suspension in water (typical concentration: 1 mg mL^−1^ and 0.5 mg mL^−1^, respectively) was added to 2.5 mL TlCl aqueous solution (2000 mg L^−1^) and incubated at room temperature for 3 h. The resulting Tl-PBNPs suspension was purified by ultrafiltration using Amicon® filter tubes (0.5 mL 10 000 MWCO). The mixture was centrifuged at 12 000 rpm for 5 min and the pellet resuspended in H_2_O. This process was repeated three times. Finally, in order to obtain the dry powder, Tl-PBNPs aqueous suspension was freeze-dried at −54 °C, 0.1 mbar (LTE Scientific™ Freeze Dryer Lyotrap).

### Radiolabelling of PBNPs with ^201^Tl

The required amount of [^201^Tl]TlCl activity (30–1000 kBq) was added to the same volume (1 : 1) of CA-PBNPs or Chi-PBNPs aqueous suspension (1 mg mL^−1^ and 0.5 mg mL^−1^, respectively) and incubated at room temperature for different times (5–180 min). Chi-PBNPs were also subjected to radiolabelling at increased temperature (37–70 °C) in order to accelerate and increase the efficacy of the radiolabelling process. Radiolabelling yield (RLY) was determined by thin layer chromatography (TLC); if RE was under 90%, the ^201^Tl-PBNPs were purified by centrifugation at 12 000 rpm for 5 min using Amicon® filter tubes (0.5 mL, 10 000 MWCO) and resuspending in MilliQ water. For the TLC method, 1–3 μL of [^201^Tl]TlCl (control) and ^201^Tl-CA-PBNPs or ^201^Tl-Chi-PBNPs were spotted on 10 cm long cellulose TLC strips (Whatman), dried at room temperature and placed in mobile phase of 10 mM EDTA solution (ethylenediaminetetraacetic acid disodium salt, dihydrate). The chromatograms were analysed with a phosphor imager (Amersham Typhoon) and Amersham Typhoon Software. Rf ^201^Tl = 1, Rf ^201^Tl-PBNPs = 0.

### Radiochemical stability

50 μL of purified ^201^Tl-CA-PBNPs or ^201^Tl-Chi-PBNPs aqueous solution (concentration: 0.5 mg mL^−1^ and 0.25 mg mL^−1^, respectively) were added to 50 μL of either human serum (H4522), RPMI-1640 medium or MilliQ water and kept at room temperature or 37 °C for 24 h, 72 h and 7 days. After each time point, radiochemical stability was assessed by TLC. For assessing the stability in 0.9% NaCl and 5–150 mM KCl solutions, 50 μL of ^201^Tl-CA-PBNPs or ^201^Tl-Chi-PBNPs aqueous solution (concentration: 0.5 mg mL^−1^ and 0.25 mg mL^−1^, respectively) were centrifuged at 12 000 rpm for 5 min using Amicon® filter tubes (0.5 mL, 10 000 MWCO), resuspended with 50 μL of the tested solution and kept at room temperature for 24 h, 72 h and 7 days.

### Physicochemical characterisation

#### ζ-potential and particle size

Dynamic light scattering (DLS) size measurements were carried out using a Zetasizer Nanoseries ZS90 instrument (Malvern Instrumentals Ltd) equipped with He–Ne laser (*λ* = 633 nm). Before the analysis, CA-PBNPs and Chi-PBNPs samples (concentration: 1 mg mL^−1^ and 0.5 mg mL^−1^, respectively) were diluted 8-fold with MilliQ water and sonicated for 10 min.

#### Transmission electron microscopy (TEM)

TEM was performed to visualise the morphology of nanoparticles and assess their size, using a JOEL JEM 1400 Plus transmission electron microscope (Centre for Ultrastructural Imaging, KCL, and at Advanced Bioimaging Research Technology Platform, University of Warwick). Size distribution was measured as a diameter using ImagJ software, *n* > 100.

#### Fourier transform-infrared spectroscopy (FT-IR)

Samples in crystal and powder form were analysed, using either a Bruker Alpha-I FT-IR spectrometer (Bruker Optics Ltd, Coventry, UK) or a Frontier IR/NIR system (PerkinElmer). Spectra were generated at room temperature in the mid-IR region (4000–400 cm^−1^).

#### Thermogravimetric analysis (TGA)

TGA was carried out at the Polymer Characterisation Research Technology Platform, University of Warwick. The TGA data were recorded on a Mettler Toledo TGA/DSC 1 instrument, in a nitrogen atmosphere flowing at 50 mL min^−1^. The samples were prepared in 70 μL pans and subjected to a temperature profile of 40–800 °C at a ramp rate of 20 °C min^−1^.

#### Elemental analysis (EA)

EA of CA-PBNPs and Tl-CA-PBNPs as freeze-dried powder was performed by Medac Ltd (Chertsey Road, Chobham, GU24 8JB, UK). Carbon, hydrogen and nitrogen mass percentage (wt%) was determined by the quantitative dynamic flash combustion method using the FlashEA® 1112 Elemental Analyzer. The instrument was calibrated with the analysis of standard compounds using the linear regression method incorporated in the EAGER300™ software. For the thallium and iron analysis, inductively coupled plasma-optical emission spectroscopy analysis (ICP-OES, Varian Vista MPX ICP-OES system) was performed. The sample was digested with nitric acid and sulfuric acid on a hotplate. A calibration curve was prepared using serial dilutions of the standard solution. The concentration of metal in the sample solution was calculated by running the sample solution against the calibration curve.

#### X-ray diffraction (XRD)

XRD measurements were made using an Anton Paar XRDynamic 500 equipped with a Primux 3000 X-ray tube giving Co K_α1,2_ radiation (*λ* = 1.7902 Å) and a Pixos 2000 1D detector. The CA-PBNPs samples were measured in reflection while Chi-PBNPs samples were held between Kapton windows and measured in transmission mode. Measurements were made in the range 15–70° 2*θ* in reflection and 5–50° in transmission with a step size of 0.02°. Rietveld refinements of CA-PBNPs and Tl-CA-PBNPs were made using Topas Academic 6.0 with a starting model taken from ICSD entry 23102.^28^

#### Small-angle X-ray scattering (SAXS)

Small-angle X-ray scattering (SAXS) measurements were made using a Xenocs Xeuss 2.0 equipped with a micro-focus Cu Kα source collimated with Scatterless slits. The scattering was measured using a Pilatus 300 k detector with a pixel size of 0.172 mm × 0.172 mm. The distance between the detector and the sample was calibrated using silver behenate (AgC_22_H_43_O_2_), giving a value of 2.498(2) m. The magnitude of the scattering vector (*q*) is given by *q* = (4π sin *θ*)/*λ*, where 2*θ* is the angle between the incident and scattered X-rays and *λ* is the wavelength of the incident X-rays. This gave a *q* range for the detector of 0.0046 Å^−1^ and 0.15 Å^−1^. An azimuthal integration of the 2D scattering profile was performed using Xenocs Xsact software and the resulting data corrected for the absorption, sample thickness and background.^29^ Samples were mounted in 1 mm thick borosilicate glass capillaries and each sample was measured for 4 hours. The measured SAXS data were modelled utilising the Monte Carlo fitting approach of the McSAS software package.^30^ The scattering was modelled as a core–shell structure with the core radius and shell thickness modelled as size distributions. For the modelling purposes, it was assumed the shell was made of 50% H_2_O and 50% citric acid or chitosan.^31^ The chemical formula and density from the Rietveld refinement results were used on all samples.

#### X-ray absorption near edge structure (XANES)

XANES data for the PB materials were collected using the easyXAFS300+ spectrometer in transmission mode, with the Fe K-edge energy range accessed using a Si 531 spherically bent crystal analyser.^32^ A helium gas chamber was used in the X-ray flight path to reduce air scattering. Measurements were made in the pre-edge, XANES and EXAFS according to Table S4 (ESI†). Sample weights of PB reference, CA-PBNPs, Tl-CA-PBNPs, Chi-PBNPs, and Tl-Chi-PBNPs were 13.63, 17.10, 18.85, 11.31, and 12.33 mg respectively. Samples were pressed into 13 mm diameter pellets using cellulose (15 mg). The raw data were dead-time corrected, normalised (using the empty beam) and energy calibrated (using metallic Fe foil) with the instrument software. The subsequent pre-edge background subtraction and post-edge normalisation were carried out using Athena software.^33^

#### X-ray absorption fine structure (EXAFS)

The normalised spectra in the EXAFS energy region (roughly 50 eV above the Fe K-edge reference energy) were first converted into k space with a *k*^2^-weighted enhancement to make the spectrum intensity more pronounced at higher *k* values. Fourier transforming the *k*^2^-weighted data set in the *k* range of 3.3–11 Å^−1^ enabled model peak fitting in the *R* space along the range of 1–3.0 Å. An experimental crystal model served as the starting model to simulate the PB multipath EXAFS legs generated *via ab initio* multiple scattering calculations of X-ray absorption matter events encoded in the FEFF9 code.^34,35^ Artemis software was used to perform the multipath fitting of the experimental EXAFS region so that Fe–C and Fe–N bond lengths were resolved to a precision of 0.01 Å.^33^

#### X-ray fluorescence (XRF)

XRF measurements were made on a 4 kW Rigaku Primus IV Wavelength Dispersive XRF instrument. Material weight of PB reference, CA-PBNPs, Tl-CA-PBNPs, Chi-PBNPs, and Tl-Chi-PBNPs were 13.63, 17.10, 18.85, 11.31, and 12.33 mg respectively. The samples were pressed into 13 mm diameter pellets using cellulose (15 mg) and reused after XANES and EXAFS measurements after removal of both the Kapton tape and the top surface of the pellet. The pellets were measured using a 10 mm mask and samples were weighed to allow a weight/thickness correction. Qualitative “standardless” scans were made to detect elements from boron to uranium. A fundamental parameters model using standards measured in the factory was used to model the matrix and allow determination of the composition of the sample. The Si-based adhesive on the Kapton used in the XANES measurements was the source of the additional Si content. The method's margin of error is estimated to fall within a range of ± 5% relative to the specified mass percentage.

#### Statistical analysis

Data were analysed in GraphPad Prism 9.1.0, and expressed as mean ± SD.

## Author contributions

K. M. W. conceived, designed and conducted the experiments and wrote the manuscript and the subsequent revisions. P. J. B. conceived the project, supervised the experimental section and reviewed the manuscript. G. P.- F. conducted the XANES and EXAFS experiments, analysed the data and reviewed the manuscript. S. H. conducted the XRD experiments, analysed the data and reviewed the manuscript. RTMdR supervised the experimental section and reviewed the manuscript. D. W. conducted the SAXS and XRF experiments, supervised the XRD experiments, analysed the data and reviewed the manuscript. S. Y. T. supervised the project and reviewed the manuscript. V. A. supervised the project and reviewed the manuscript. J. P. conceived the project, designed and supervised the experimental section, supervised the preparation of the manuscript and reviewed the manuscript and subsequent revisions.

## Data availability

The data supporting the findings of this study are available within the article and its ESI.†

## Conflicts of interest

There are no conflicts of interest to declare.
